# Distribution of Hydrogen-Producing Bacteria in Tibetan Hot Springs, China

**DOI:** 10.3389/fmicb.2021.569020

**Published:** 2021-07-21

**Authors:** Li Ma, Geng Wu, Jian Yang, Liuqin Huang, Dorji Phurbu, Wen-Jun Li, Hongchen Jiang

**Affiliations:** ^1^State Key Laboratory of Biogeology and Environmental Geology, China University of Geosciences, Wuhan, China; ^2^Tibet Plateau Institute of Biology, Lhasa, China; ^3^State Key Laboratory of Biocontrol, Guangdong Key Laboratory of Plant Resources, School of Life Sciences, Sun Yat-sen University, Guangzhou, China

**Keywords:** Tibet, hot springs, hydrogen-producing bacteria, [FeFe]-hydrogenase, temperature

## Abstract

Investigating the distribution of hydrogen-producing bacteria (HPB) is of great significance to understanding the source of biological hydrogen production in geothermal environments. Here, we explored the compositions of HPB populations in the sediments of hot springs from the Daggyai, Quzhuomu, Quseyongba, and Moluojiang geothermal zones on the Tibetan Plateau, with the use of Illumina MiSeq high-throughput sequencing of 16S rRNA genes and *hydA* genes. In the present study, the *hydA* genes were successfully amplified from the hot springs with a temperature of 46–87°C. The *hydA* gene phylogenetic analysis showed that the top three phyla of the HPB populations were *Bacteroidetes* (14.48%), *Spirochaetes* (14.12%), and *Thermotogae* (10.45%), while *Proteobacteria* were absent in the top 10 of the HPB populations, although *Proteobacteria* were dominant in the 16S rRNA gene sequences. Canonical correspondence analysis results indicate that the HPB community structure in the studied Tibetan hot springs was correlated with various environmental factors, such as temperature, pH, and elevation. The HPB community structure also showed a spatial distribution pattern; samples from the same area showed similar community structures. Furthermore, one HPB isolate affiliated with *Firmicutes* was obtained and demonstrated the capacity of hydrogen production. These results are important for us to understand the distribution and function of HPB in hot springs.

## Introduction

Studying microbial distribution and function in geothermal environments is of great significance to the understanding of life evolution and the biogeochemical cycle of elements in high-temperature environments. Geothermal environments are characterized by high temperature, oligotrophic conditions, low dissolved oxygen, and high concentrations of hydrogen gas (Xu and Glansdorff, 2002; Konhauser et al., 2003; Zgonnik, 2020). Hydrogen can serve as one electron donor for microorganisms in geothermal environments (Lindsay et al., 2018; Gregory et al., 2019). The utilization of hydrogen is one of the oldest and most basic properties of life (Kasting, 1993; Lepot, 2020), such that a large quantity of prokaryotes use hydrogen as one energy source (Schwartz and Friedrich, 2006; Fedonkin, 2009). Hydrogen in geothermal environments can be formed through geological (abiotic) and biological (biotic) processes (Brazelton et al., 2011; Smetannikov, 2011). The abiotic formation of hydrogen such as the interaction between water and crustal iron minerals has been reported in hydrothermal fluids (Lindsay et al., 2019). However, less attention has been given to the biotic formation of hydrogen in geothermal environments.

In biological processes, fermentative bacteria transform and degrade organic matter and produce metabolic substrates under anaerobic conditions, such as hydrogen, carbon dioxide, and organic acids. Hydrogenase is one key enzyme in the process of microbial hydrogen production. Indeed, about 30% of microorganisms have genes encoding hydrogenases (Peters et al., 2015). Hydrogenase can be divided into [FeFe]-, [NiFe]-, and [Fe]-hydrogenase according to the metal type of their binding sites (Tard and Pickett, 2009; Lubitz et al., 2014; Greening et al., 2016; Schilter et al., 2016). Generally, [NiFe]-hydrogenase, a type of hydrogen-absorbing enzyme, generally exists in microorganisms that consume hydrogen, but some of them were recently discovered to be present in microorganisms that produce hydrogen; [Fe]-hydrogenase is found only in archaea. In contrast, [FeFe]-hydrogenase catalyzes the reduction of protons to form hydrogen in the presence of electron donors and low oxidation potentials and plays an important role in the formation of hydrogen, but that they may also be reversible, or tied to H_2_ consumption (e.g., as in electron bifurcation) (Vignais et al., 2001; Vignais and Billoud, 2007; Avilan et al., 2018; Schuchmann et al., 2018). The large subunit of the [FeFe]-hydrogenase is encoded by the *hydA* gene that is relatively conserved. So, the *hydA* gene is used for an effective molecular marker to examine the distribution of hydrogen-producing bacteria (HPB) in different environments such as sewage (Tomazetto and Oliveira, 2013), paddy soil (Baba et al., 2014), marine hot springs (Xu et al., 2013), limestone sinkholes (Sahl et al., 2011), terrestrial hot springs (Boyd et al., 2010), anaerobic bioreactor, and salt lakes (Defeng et al., 2008; Boyd et al., 2014). Among these results, *hydA* genes were mainly affiliated with *Thermotogae*, *Firmicutes*, *Proteobacteria*, and *Bacteroidetes*. Nevertheless, previous studies were based on cloning and sequencing methods, the depth of information acquisition was insufficient, and they rarely involved the impact of environmental factors (e.g., temperature) on the hydrogen-producing microbial community. As one of the important environmental factors in the geothermal environment (Skirnisdottir et al., 2000; Pearson et al., 2008), temperature also plays an important role in the biological hydrogen production process (Spear et al., 2005; Baghchehsaraee et al., 2008; Zheng et al., 2014). However, little is known about how temperature and other factors affect the diversity of hydrogen-producing microorganisms in geothermal features.

Tibetan hot springs are generally located at an elevation of over 4,000 m and receive fewer human perturbations, with a wide range of temperature from 22 to 94°C. Previous studies have investigated the compositions of total microbial communities and some functional microbial groups and their response to environmental variables in Tibetan hot springs (Lau et al., 2006, 2009; Lau and Pointing, 2009; Huang et al., 2011; Song et al., 2013; Wang et al., 2013; Wu et al., 2015; Yang et al., 2015; Zhang et al., 2018; Ma et al., 2021). The purpose of this study was to investigate the diversity of the HPB and their response to environmental factors in Tibetan hot springs.

## Materials and Methods

### Sample Collection

**FIGURE 1:**
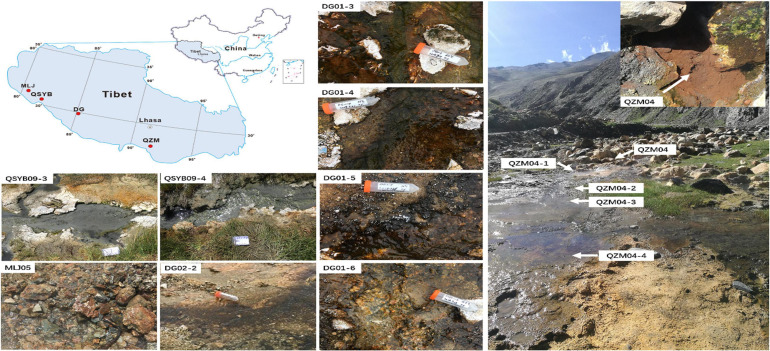
The locations of the studied geothermal zones in the Tibetan Plateau. QSYB, Quseyongba; MLJ, Moluojiang; DG, Daggyai; and QZM, Quzhuomu.

In the present study, *hydA* gene was successfully amplified in a total of 13 samples from 66 Tibetan hot spring samples (Supplementary Figure 1), including nine samples from two outflowing channels in the Tibetan hot springs (Figure 1). In the Daggyai geothermal zone, the five sampled individual hot springs were labeled with DG01-3, –4, –5, –6, and DG02-2, covering a temperature range of 46–63°C; In the Quzhuomu geothermal zone, the collected samples along the outflowing channels of hot springs were labeled with QZM04, 04-1, –2, –3, -and –4, covering a temperature range of 54–72°C. In addition, three sample hot springs were selected in the Moluojiang geothermal area and the Quseyongba geothermal area and were labeled with MLJ-05 (87°C), QSYB09-3 (70°C), and QSYB09-4 (66°C), respectively. At each sampling site, water temperature and pH were measured *in situ* with a temperature/pH probe (LaMotte, Chestertown, MD, United States). Water chemistry parameters, such as oxidation–reduction potential index (Eh), electrical conductivity (EC), and total dissolved solids (TDS), were measured *in situ* by using portable water quality analyzer (Thermo Scientific ORION STAR A329, Beverly, MA, United States). For dissolved organic carbon (DOC) analysis, 25 ml of hot spring water was collected by a sterile 50-ml syringe and filtered through pre-combusted (450°C, 4 h) GF/F filters (0.7-mm pore size, Whatman, Buckinghamshire, United Kingdom), and the resulting filtrate was collected into pre-combusted brown glass bottles with the addition of concentrated phosphoric acid [final concentration 0.2% (v/v)]. Hot spring water (25 ml) was filtered through 0.22-m nitrocellulose membranes and collected into acid-washed polyethylene bottles for major anion measurement; 25 ml of hot spring water was filtered through polycarbonate membrane filters (pore size 0.22 mm) and collected into glass bottles supplemented with concentrated HNO_3_ (to a final concentration of 0.1 M) for major cation measurement. Sediments for microbial analyses were collected in 50-ml Falcon tubes and were then frozen in dry ice in the field and during transportation. Once in the laboratory, the sediment samples were stored at –80°C until further analysis.

### Geochemical Analyses

Major anion concentrations of the collected hot spring water samples were measured using an ion chromatograph (IonPac AS18 4 × 250 mm for anion, ICS 600, Thermo Fisher, Carlsbad, USA). The standard stock solutions (100 μg ml^–1^) of multi-ion anion standards including fluoride (F^–^), chloride (Cl^–^), nitrate (NO_3_^–^), and sulfate (SO_4_^2–^) were purchased from General Research Institute for Non-ferrous Metals (No. GSB04-3369-2016, Beijing, China). Column temperature was maintained at 30°C, the flow rate was 1 ml/min, and eluent liquid was 31 mM NaOH. The DOC contents were measured by using an NC 2100 Elemental Analyzer (multi N/C 2100, Analytic Jena, Jena, Überlingen, Germany). The concentrations of Ca, K, Mg, Na, and Fe were determined by ICP-OES (Thermo Fisher ICAP-6300, Carlsbad, United States). The standard stock solutions (1 μg ml^–1^) of multi-element standards including sodium (Na), potassium (K), calcium (Ca), and magnesium (Mg) stored in 5% HNO_3_ were purchased from General Research Institute for Non-ferrous Metals (No. GNM-M275017-2013, Beijing, China).

### DNA Extraction, PCR Amplification, and Sequencing

Total community DNA of the collected hot spring sediments were extracted with FastDNA Spin Kit according to the manufacturer’s instructions (MP Biomedicals, LLC, Solon, OH). Bacterial and Archaeal 16S rRNA genes were amplified with a universal primer set of 515F (5′GTGYCAGCMGCCGCGGTA-3′)/806R (5′-GGACTACVSGGGTATCTAAT-3′) according to the PCR conditions described previously (Tamaki et al., 2011; Caporaso et al., 2012; Zhang et al., 2018). The *hydA* genes of the total community DNA in the collected hot spring sediments were PCR amplified using the primer set FeFe-272F (5′-GCHGAYMTBACHATWATGGARGA-3′, where H = A, C, T; Y = C, T; M = A, C; B = C, G, T; W = A, T; R = A, G; 432-fold degeneracy)/FeFe-427R (5′-GCNGCYTCCATDACDCCDCCNGT-3′, where N = A, C, T, G; Y = C, T; D = A, G, T; 864-fold degeneracy) (Boyd et al., 2010; Sahl et al., 2011; Baba et al., 2014). A unique 12-bp barcode sequence is added between the sequencing adapter and the forward primer to distinguish among samples. PCR conditions of *hydA* genes were as follows: initial denaturation at 94°C (4 min), and 35 cycles of denaturation at 94°C (1 min), annealing at 56.5°C (1 min), and extension at 72°C (1 min), followed by a final extension at 72°C (20 min) (Boyd et al., 2009, 2010, 2014). In order to ensure the reliability of the results, triplicate PCRs and experimental blanks were conducted for each sample. Then, the successful 16S rRNA gene and *hydA* gene PCR products were purified with the AxyPrep DNA Gel Extraction Kit (Axygen Scientific Inc., Union City, CA, United States) and sequenced by using an Illumina-Hiseq2500 platform (2 × 250 bp) and Illumina-MiSeq platform (2 × 300 bp), respectively.

### Processing of the 16S rRNA Gene and *hydA* Gene Illumina Sequencing Data

The obtained paired-end raw reads were quality filtered with Trimmomatic (V0.33)^1^. Reads having the following properties were removed: (1) receiving a quality score < 20; (2) containing one or more ambiguous base calls; and (3) comprising consecutive high-quality base calls < 75% of the total read coverage length. The resulting high-quality paired-end reads were assembled with FLASH (V1.2.11, fast length adjustment of short reads) using default settings (Magoc and Salzberg, 2011). Sequences were assigned to each sample based on their unique barcodes, and then the barcodes and primers were removed. The effective sequences were clustered into operational taxonomic units (OTUs) at the cutoff of 97% similarity by using the USEARCH software, and chimeric sequences and singleton OTU sequences were excluded to avoid possible biases (Edgar et al., 2011). Phylogenetic information was assigned for the obtained representative OTU sequences by using the SILVA database v132 (for 16S rRNA)^2^ and Non-Redundant Protein Sequence Database (for *hydA*)^3^ with the confidence threshold ≥ 0.5 by default. The OTU table was rarefied to an equal sequence number for each sample, and then the resulting rarefied OTU table was employed in subsequent analyses unless specified otherwise.

### Statistical Analysis

We standardized the measured environmental factor data and applied it together with the *hydA* OTU table for the subsequent statistical analysis (Table 1). Statistical analysis was performed by using various packages in the R program^4^ (version R-3.5.2, 2018-12) unless stated otherwise. Alpha diversity indices were calculated by using R package “vegan.” Linear regression analysis was employed to assess the correlation between the observed OTUs and measured environmental factors of the studied samples by using the PAST software. Relative abundance histogram was constructed to assess differences in population abundance in samples from different regions by using package “reshape2,” and Pearson’s rank correlations were calculated between the measured environmental variables and population relative abundance by using the package “Hmisc.” The autocorrelation test of environmental factors was carried out by using the package “Hmisc.” The analyzed geochemical parameters were assessed for the variable inflation factor (VIF) using the Vegan package of R Project to reduce the number of highly co-linear variables. The VIF assessment was repeated until all the remaining environmental variables exhibited maximum VIF values of no higher than 10. After removing the autocorrelation factor, canonical correspondence analysis (CCA) was applied to evaluate the impact of remaining environmental factors and spatial variables on the HPB community structures by using “vegan” and “ggplot2” packages. A spatial decomposition method, principal coordinates of neighbor matrices (PCNM), was applied to the geographic coordinates of the samples. This method separates sample geographic coordinates into multiple spatial variables. Then, the significant (*p* < 0.05) spatial variables were selected for variation partitioning analysis (VPA). VPA was performed to determine the relative contributions of the measured environmental variables (environmental factors after removing autocorrelation) and spatial variables to the distribution of HPB community structures using the “vegan” package. The residual proportion represented unexplained variance.

**TABLE 1:** Geographical and geochemical parameters of the investigated hot springs in this study.

**Sites**	**Characteristics**	**Samples**	**GPS location (N/E)**	**Elevation (m)**	**pH**	**Temp (°C)**	**Eh (mv)**	**DOC (mg/L)**	**EC (μs/cm)**	**TDS (mg/L)**	**Fe**
Quseyongba (QSYB)	Channel springs	QSYB09-3	81.5806°/30.5848°	4,620	8.9	70	–278.8	28.38	1,828	896.3	2.13
		QSYB09-4	81.5806°/30.5848°	4,620	8.96	66	–274.1	12.11	1,804	884.2	1.84
Moluojiang (MLJ)	Individual spring	MLJ-05	80.4914°/31.3690°	4,863	8.48	87	–94.8	1.86	5,447	2670	1.16
Daggyai (DG)	Channel springs	DG01-3	85.7509°/29.5982°	5,057	8.7	60	–102.9	21.86	1,834	899.3	0.86
		DG01-4	85.7509°/29.5982°	5,057	8.71	55	–104.7	8.05	1,855	909.6	0.96
		DG01-5	85.7509°/29.5982°	5,057	8.75	50	–106.4	18.81	1,840	902.3	1.11
		DG01-6	85.7509°/29.5982°	5,057	8.6	46	–101.9	23.45	1,835	899.8	0.92
	Individual spring	DG02-2	85.7507°/29.5988°	5,058	8.55	63	–102.6	8.03	1,842	903.1	0.92
Quzhuomu (QZM)	Channel springs	QZM04	91.8127°/28.2367°	4,853	6.87	72	–0.3	18.26	2,037	998.4	2.38
		QZM04-1	91.8127°/28.2367°	4,853	7.17	67	–18.0	18.27	2,057	1,025	1.88
		QZM04-2	91.8127°/28.2367°	4,853	6.98	62	–6.3	21.01	2,085	1,022	2.09
		QZM04-3	91.8127°/28.2367°	4,853	7.29	58	–24.7	21.8	2,082	1,021	1.79
		QZM04-4	91.8127°/28.2367°	4,853	7.6	54	–42.5	11.82	2,054	1,007	1.37

### Cultivation and Isolation of HPB

The hot spring sediments of QZM04-4 with positive *hydA* gene amplification were chosen for HPB isolation. Previous studies showed that hydrogen production is the most effective after heat treatment at 55°C (Zheng et al., 2014), which covered the samples in the main temperature range of our study. So, the QZM04-4 hot spring sediment was selected for HPB isolation. One milliliter of sediment slurry (hot spring sediment:water = 1:30, v:v) was inoculated into pre-reduced PY broth in glove box (Holdeman et al., 1977). The basal medium used for cultivation of HPB contained the following (per liter): 0.5 g of KH_2_PO_4_, 1.2 g of Na_2_HPO_4_, 0.29 g of NH_4_Cl, 0.29 g of NaCl, 0.001 g of CaCl_2_⋅2H_2_O, 0.01 g of MgCl_2_⋅6H_2_O, 1 g of yeast extract, 0.05 g of cysteine-HCl, 1 ml of 0.1% resazurin solution, and 10 ml of trace mineral element solution (Mei et al., 2014). After sterilization, glucose was added as substrate in concentration (2 g/L, final concentration), and NaHCO_3_ (3.92 g/L, final concentration) and Na_2_S⋅9H_2_O (0.02 g/L, final concentration) were added as reducing agents. Then, the cultures were cultivated at 55°C for 24 h. Subsequently, the resulting enrichments were used for isolation with the use of Hungate roll-tube technique (Hungate, 1969). Single colonies were selected and transferred to the same broth, followed by incubation at 55°C for 3 days. The cultures were added with 0.2% BrES to inhibit the growth of methanogens. The above isolation procedures were repeated several times until pure cultures were obtained.

### DNA Isolation, PCR Amplification, and Phylogenetic Analyses of Isolates

Genomic DNA of the obtained bacterial isolates was extracted with FastDNA Spin Kit (MP Biomedicals, LLC, Solon, OH). The 16S rRNA and *hydA* genes of the obtained isolates were PCR amplified using the primer sets of 27F/1492R (Frank et al., 2008) and FeFe-272F/FeFe-427R, respectively. PCR conditions of 16S rRNA genes were as follows: initial denaturation at 95°C (5 min), and 35 cycles of denaturation at 94°C (30 s), annealing at 52°C (30 s), and extension at 72°C (1.5 min), followed by a final extension at 72°C (10 min). PCR conditions of *hydA* genes were the same as those of the sediment samples. The resulting successful 16S rRNA and *hydA* gene PCR products were purified with the AxyPrep DNA Gel Extraction Kit. The purified 16S rRNA and *hydA* gene PCR products of strain QZM-1 were sequenced with Sanger sequencing. The obtained sequences were aligned in GenBank database (version 242). Maximum likelihood phylogenetic trees of the 16S rRNA sequence and *hydA* amino acid sequence of the obtained bacterial isolates were constructed with the Poisson model in MEGA 7.0 (Saitou and Nei, 1987) and bootstrap replicates 1,000 times.

### Hydrogen Production Test of Hydrogen-Generating Cultures

The H_2_ production tests were performed under batch conditions in 120-ml serum bottles capped with rubber stoppers. Three milliliters of the early-stationary-phase pre-culture on PY medium (containing 2 g/L of glucose) was transferred anaerobically in 30 ml of the basal culture medium as inoculum. Prior to inoculation, the upper air and lower medium liquid go through N_2_-flushing and sterilization. The experiment was carried out at 55°C and assays were performed at least in triplicate. One milliliter of the headspace was regularly (4, 8, 12, 24, 48, and 96 h) collected for measuring H_2_ content with the use of one Agilent 7890A gas chromatograph with a TCD detector, the column model was C38743-14, carrier gas was nitrogen at a pressure of 100 kPa, and running time was 2 min.

### Nucleotide Sequence Accession Numbers

The 16S rRNA high-throughput sequences generated in this study were deposited in the NCBI SRA database under the BioProject ID PRJNA638734 with accession number SRR12042849–SRR12042861. The *hydA* high-throughput sequences generated in this study were deposited in the NCBI SRA database under the BioProject ID PRJNA693970 with accession number SRR13505064–SRR13505076.

## Results

### Geochemical Characteristics of the Studied Hot Springs

Geochemical characteristics of the studied hot springs are diverse (Table 1). Briefly, the elevation of the studied hot spring ranged from 4,620 m above sea level (masl) to 5,057 masl; the temperature of the studied hot springs ranged from 46 to 87°C. The hot springs in the Quzhuomu geothermal zone possessed lower pH (6.8–7.6) than those in the other geothermal zone (8.55–8.96), while Eh index (–0.3 to –42.5) was higher than other geothermal zones (–94.8 to –278.8). The values of EC and TDS in the Moluojiang geothermal zone were the highest, while those in other areas were more consistent (Table 1). In addition, hot springs from the Quzhuomu geothermal zone had higher concentrations of Ca^2+^ (163.7–173.0 mg L^–1^ vs. 0.97–2.02 mg L^–1^) and SO_4_^2–^ (491.2–513.7 mg L^–1^ vs. 87.17–90.18 mg L^–1^) than those from the DG geothermal zones, while the concentration of Na^+^ (297.6–315.4 mg L^–1^ vs. 166.8-172.2 mg L^–1^) in the DG area was higher than that in the Quzhuomu area. From the results of the Cl^–^/SO_4_^2–^ ratio, the concentration of Cl^–^ was higher than that of SO_4_^2–^ in the MLJ, QSYB, and DG geothermal areas, while the results of the QZM geothermal area were just the opposite. This suggested that only the QZM hot spring was mainly influenced by vapor phase, while hot springs in other areas were mainly influenced by liquid phase.

### 16S rRNA and *hydA* Gene Diversity of the Studied Hot Springs

**FIGURE 2:**
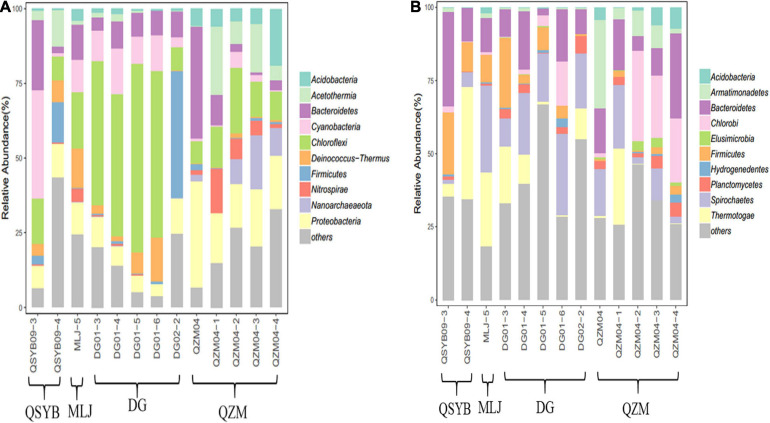
Phylogenetic analysis of the obtained 16S rRNA **(A)** and *hydA* gene **(B)** sequences of the studied hot spring samples.

**FIGURE 3:**
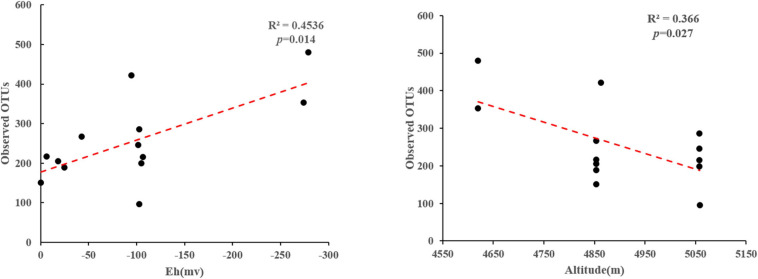
Linear regression analyses showing correlations between the observed OTUs and measured environmental factors of the studied samples.

Phylogenetic analysis showed that these 16S rRNA gene OTUs were mainly affiliated with the 10 most dominant phyla, i.e., *Chloroflexi* (25.30%), *Proteobacteria* (13.15%), *Bacteroidetes* (10.00%), *Cyanobacteria* (8.23%), *Acetothermia* (5.65%), *Firmicutes* (5.08%), *Deinococcus-Thermus* (4.13%), *Acidobacteria* (3.90%), *Nanoarchaeaeota* (3.01%), and *Nitrospirae* (2.77%), as well as others (18.77%), in a decreasing order (Figure 2A). A total of 771,199 high-quality *hydA* gene sequences were obtained from all the studied hot spring samples (*n* = 13), with an average of 59,323 sequences per sample. These sequences were classified into 3331 OTUs (Supplementary Table 1). The observed OTUs were significantly (*p* < 0.05) negatively correlated with Eh and elevation (Figure 3). Phylogenetic analysis showed that these *hydA* gene OTUs were mainly affiliated with the 10 most dominant phyla, i.e., *Bacteroidetes* (14.48%), *Spirochaetes* (14.12%), *Thermotogae* (10.45%), *Chlorobi* (7.66%), *Firmicutes* (6.93%), *Armatimonadetes* (4.51%), *Planctomycetes* (2.45%), *Acidobacteria* (1.63%), *Hydrogenedentes* (0.75%), and *Elusimicrobia* (0.68%), as well as others (36.34%), in a decreasing order (Figure 2B).

The *hydA* genes affiliated with *Bacteroidetes* and *Spirobacteria* dominated the studied samples regardless of their temperature or regions, but the abundances of other phyla were different. For example, the *hydA* gene sequences affiliated with *Firmicutes* in the DG region showed much higher abundance than that in the QZM region (8% vs. 1.72%), while those affiliated with *Chlorob*i showed an opposite trend between these two regions (4.16% vs. 15.16%). In addition, the samples of different temperature ranges were also different. For example, in the samples with temperature higher than 70°C, the top three most abundant phyla of *hydA* genes were *Bacteroidetes*, *Spirochaetes*, and *Armatimonadetes*; those in the samples with temperature 60–70°C were *Bacteroidetes*, *Spirochaetes*, and *Thermotogae*; and those in the samples with temperature below 60°C were *Bacteroidetes, Spirochaetes*, and *Chlorobi* (Figure 2B). Pearson’s rank correlation analysis showed that the relative abundance of *hydA* genes affiliated with *Firmicutes* was significantly positively correlated with pH (*p* < 0.05), while it was significantly negatively correlated with Eh (*p* < 0.05). In addition, the relative abundance of *hydA* genes affiliated with *Armatimonadetes*, *Acidobacteria*, and *Elusimicrobia* showed significantly negative correlation with pH (*p* < 0.05) (Supplementary Figure 2).

### Statistical Analysis

**FIGURE 4:**
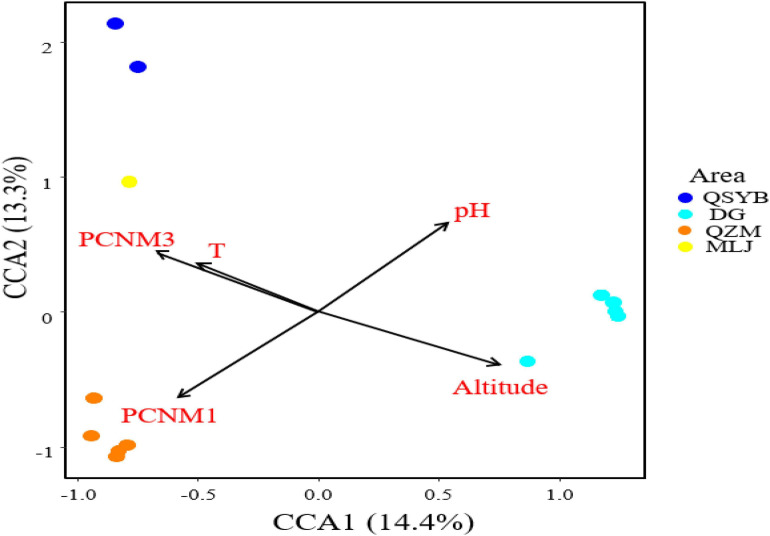
CCA analysis showing the influence of environmental factors and geographic factors on the hydrogen-producing bacteria community structures. Assemblages sampled from the QZM, DG, DG01 (DG02-2), QSYB, and MLJ area are denoted in orange, sky blue, green, dark blue, and yellow, respectively.

**FIGURE 5:**
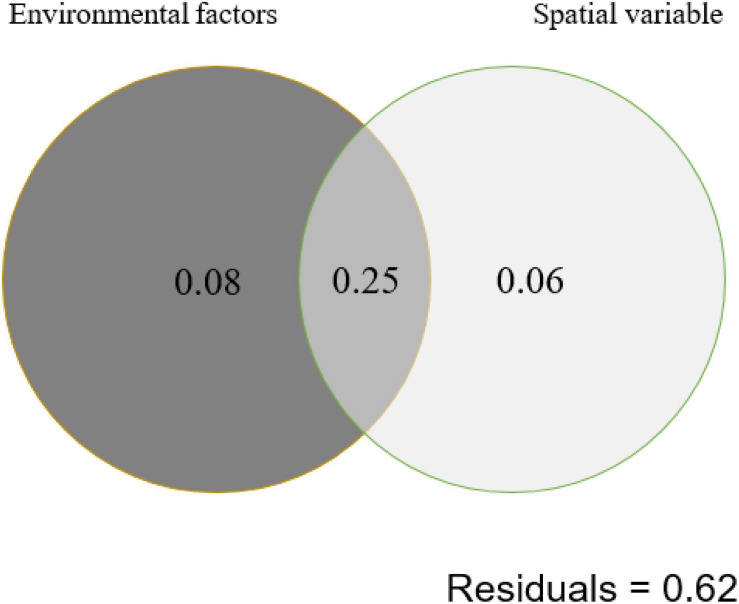
Variation partition analysis (VPA) showing the contribution of environmental and spatial factors to shaping of the *hydA* gene diversity (at the OTU level) in the studied hot spring samples. Environmental factors contain elevation, pH, and temperature. Spatial variable contains PCNM1 and PCNM3.

Autocorrelation test among the measured environmental factors showed that temperature was significantly (*p* < 0.05) positively correlated with the electrical conductivity and total dissolved solids, while the pH of the studied samples showed negative correlation with the Eh (Supplementary Figure 3). Among all the measured environmental factors, the CCA results showed that the HPB community structure was correlated with elevation, temperature, pH, and spatial factors (PCNM1 and PCNM3) (Figure 4). The VPA results showed that the measured environmental factors (e.g., elevation, pH, and temperature) and spatial variables can totally explain 39% of the observed variations of the HPB community structure in the studied hot springs. These two types of factors (i.e., environmental vs. spatial) co-explained 25% variations, while they accounted for 33% and 31% of the observed variations of the HPB community structure, respectively (Figure 5).

### Phylogenetic Analysis and Hydrogen Production of Strain QZM-1

One strictly anaerobic hydrogen-producing bacterium was isolated from the sediments of Quzhuomu hot spring and was named QZM-1. The full length of the 16S rDNA sequence of strain QZM-1 was 1,424 bp. Phylogenetic analysis showed that QZM-1 is closely related to *Thermobrachium celere* and *Caloramator coolhaasii*, with 16S rRNA gene similarity of 98.38% and 97.47%, respectively. The *hydA* gene sequence of strain QZM-1 showed 98.58% similarity with *T. celere.* Therefore, the phylogenetic analysis suggested that strain QZM-1 likely belonged to the genus *Caloramator* rather than *Thermobrachium* (Supplementary Figures 4A,B). Our high-throughput data showed that the sequence reads belonging to the *Caloramator* genus accounted for 0.5 and 0.37% of the total 16S rRNA and *hydA* genes, respectively. Strain QZM-1 showed the capacity of producing hydrogen in the fermentation experiments (Supplementary Figure 5). Hydrogen production occurred at the time of the first sampling point. After the logarithmic growth phase, the hydrogen production gradually stabilized and reached the maximum value of 3.32% H_2_ at 96 h (Supplementary Figure 5). The GenBank accession numbers of 16S rRNA and *hydA* genes of strain QZM-1 were MW517843 and MW514309, respectively.

## Discussion

### HPB Diversity in the Tibetan Hot Springs

In this study, the HPB populations in the Tibetan hot spring samples were mainly composed of species affiliated with *Bacteroidetes* and *Spirochaetales*, similar to the dominant species in neutral high-temperature oil wells (Liu et al., 2016), but different from the dominant species in acidic hot springs in Yellowstone National Park (YNP) (Boyd et al., 2010). *Proteobacteria*-related HPB have been repeatedly found to be the main group in acidic and neutral environments. It is worth noting that *Proteobacteria* accounted for 13.15% of the total 16S rRNA gene sequences in the studied samples, but *Proteobacteria*-related HPB only accounted for 0.06% of the total *hydA* gene sequences. However, they were dominant in acidic and neutral environments (Boyd et al., 2010; Schmidt et al., 2010; Tomazetto and Oliveira, 2013). Such inconsistency could be ascribed to the different environmental conditions between the Tibetan hot springs and those referred hot springs or high-temperature habitats. For example, the abovementioned oil reservoir environment had similar temperature and pH conditions to the hot spring samples in this study, while the temperature of the referred YNP hot springs was mostly below 50°C and that of other acidic and neutral environments was generally below 30°C. Moreover, previous studies have found that pH is a key factor affecting the HPB diversity (Boyd et al., 2010; Schmidt et al., 2010). Therefore, it is reasonable to observe the difference of the HPB populations between the studied Tibetan hot springs and other environments.

By combining the phylogenetic analysis results of 16S rRNA genes, *Spirochaetales*, *Thermotogae*, and *Chlorobi* were all the main groups containing *hydA* gene in the studied samples, but it appeared that all of these three groups occupied extremely low abundance. This finding implied that some rare populations in the environment may contribute more to the element cycle of the entire ecosystem. When we explore the relationship between microorganisms and environments, rare taxa cannot be ignored (Zhang et al., 2018).

### Environmental Factors Influencing HPB Population in the Tibetan Hot Springs

Temperature is one of the important environmental factors affecting hot spring microorganisms (Wang et al., 2013; Guo et al., 2020). For HPB, temperature is an important factor affecting the hydrogen production process (Guo et al., 2010; Ghimire et al., 2015). It has been shown that the *hydA* gene has not been found in the samples above 65°C in the YNP hot springs (Boyd et al., 2010). Interestingly, we successfully amplified the *hydA* gene in one hot spring with 87°C and in other four hot springs with temperatures > 65°, which deserved further study (Supplementary Figure 1). In general, the *hydA* gene was found more frequently in Tibetan hot springs with a temperature of 50–70°C; this also implies the suitable temperature range of microbial hydrogen production in the hot spring environment. In the studied samples, the abundances of *hydA* genes affiliated with HPB dominant groups were also different in different temperature ranges. Interestingly, in the samples with temperature above 60°C, the *hydA* gene affiliated with *Thermotogae* was more abundant than that belonging to *Chlorobi*, but in the samples with temperature below 60°C, the trend was opposite. This situation also happened in the samples from the same area. For example, in the samples of DG01-3 and DG02-2 in the DG region, the abundance of *hydA* gene affiliated with *Thermotogae* was higher than that of the other three samples, while that affiliated with *Chlorobi* showed an opposite trend. The similar situation was also found in the QZM area (Figure 2B). Therefore, such difference could be explained by the fact that different types of strains had different adaptation temperatures. *Thermotogae* were characteristic of thermophilicity, and *Chlorobi* were a type of photosynthetic autotrophic bacteria that can grow in a lower temperature range (Iino et al., 2010; Bhandari and Gupta, 2014). Such a trend was not found in the 16S rRNA gene results, suggesting that temperature may specifically affect microorganisms containing the *hydA* gene. Therefore, it is reasonable to observe from the CCA results that temperature was related with the HPB community structure in Tibetan hot springs.

pH was also one key factor affecting the population structure of HPB (Figure 4), which was consistent with previous research results. The pH variations can cause changes in the metabolic pathways of HPB, which were due to changes in the composition of dominant species (Horiuchi et al., 2002). In the present study, 4 of the 10 most dominant phyla of *hydA* genes were correlated with pH. Moreover, pH showed negative correlation with Eh in this study (Supplementary Figure 3), which also implied that Eh may play a certain role in affecting the HPB population structure. The fermentation reaction was really prevalent in the lower redox potential zone (Cammack and Robson, 2001; Jakobsen and Cold, 2007; Vignais and Billoud, 2007). So, it is reasonable that the observed HPB OTUs were negatively correlated with Eh in this study. Therefore, pH may directly or indirectly (i.e., by affecting one geochemical variable) affect the composition of HPB populations in the Tibetan hot springs.

Elevation reflected the difference between geographical and environmental factors. The elevations of the four geothermal areas in this study were different. Similarly, changes in environmental indicators caused by elevation, such as dissolved oxygen and hydrogen partial pressure, cannot be ignored either. Therefore, this result also suggests that, in addition to environmental factors, the influence of spatial variables on HPB community structure should also be considered.

### Spatial Variables Influencing HPB Population in the Tibetan Hot Springs

The HPB populations in the studied Tibetan hot springs exhibited a spatial pattern, i.e., the HPB populations in the samples from the same area were more similar, implying that spatial variables affected HPB distribution among regions. Interestingly, the co-explanation of spatial variables and environmental factors reached 25% (Figure 5). Among the studied Tibetan hot springs, spatial variables caused the difference in environmental conditions, which further jointly affected the HPB distribution. The pH of the Tibetan hot springs is generally neutral and alkaline. Only a few acidic hot springs were ever found in the DG area, while no acid hot spring was ever reported in the QZM area (Liu et al., 2019). Therefore, the DG area likely belonged to the magmatic systems and differed from those in the QZM area (Liu et al., 2019). In addition, among the studied hot spring samples, the Cl^–^/SO_4_^2–^ ratio in the DG area was higher than that in the QZM area (Table 2), which suggested that the hot springs in the DG area were more affected by liquid phase input, while the QZM area was more affected by gas phase input (Amenabar and Boyd, 2019). Furthermore, it is well known that most chemical components in geothermal water are primarily derived from its interactions with host reservoir rocks. The lithology of the reservoir host rocks in the DG area is basically granite, while the QZM area consists of marine carbonate minerals. Such lithology difference makes the hydrochemical type in the DG and QZM belong to Na-HCO_3_-Cl and Ca-Na-SO_4_-Cl, respectively (Guo et al., 2014, 2019; Liu et al., 2019; Li et al., 2020). Therefore, it is reasonable that the geological processes affect both environmental difference and the observed spatial variables causing the HPB differences in the studied Tibetan hot springs.

**TABLE 2:** Concentrations of major ions of the investigated hot springs in present study.

	**(mg L^–1^)**							
**Samples**	**Na^+^**	**K^+^**	**Ca^2+^**	**Mg^2+^**	**F^–^**	**Cl^–^**	**NO_3_^–^**	**SO_4_^2–^**
QSYB09-3	381.83	28.72	0.13	0.90	11.63	139.71	0.53	67.72
QSYB09-4	394.37	28.18	0.42	0.55	11.83	142.27	0.13	60.25
MLJ05	719.92	82.95	7.16	1.35	1.73	618.46	0.00	17.08
DG01-3	315.37	25.05	0.97	0.05	26.89	185.17	2.25	87.17
DG01-4	308.07	26.08	1.21	0.23	26.84	185.82	1.93	87.95
DG01-5	297.56	27.15	0.86	0.12	27.33	187.38	1.59	88.44
DG01-6	312.53	25.84	2.02	0.13	27.81	191.31	1.85	90.18
DG02-2	303.05	24.78	0.99	0.16	27.05	186.08	1.67	88.28
QZM04	166.81	22.26	171.29	28.26	2.60	286.06	0.03	491.20
QZM04-1	167.04	20.70	170.30	26.45	2.68	291.13	0.03	499.79
QZM04-2	169.02	21.78	172.99	28.58	2.67	295.98	0.00	512.65
QZM04-3	172.20	23.14	169.89	28.11	2.52	272.29	0.00	472.11
QZM04-4	167.00	23.24	163.71	28.72	2.70	293.60	0.35	513.69

### *hydA* Gene Served as a Biomarker for Hydrogen Production Bacteria Exploration

The functional genes were often used to explore the distribution and diversity of microbial functional groups (Yang et al., 2013; Jiang et al., 2014; Wu et al., 2015). In the present study, the hot spring sediments with positive PCR amplification of *hydA* genes were selected for the HPB isolation. Pure HPB culture was successfully obtained from the corresponding sediment samples, and the resulting pure HPB culture was successfully amplified for the *hydA* gene, followed by verifications for the function of hydrogen production and by the phylogenetic identification consistency between the resulting pure HPB culture and the 16S rRNA gene sequences from the same sediment samples. These results provided a new way of isolation and purification of functional microorganisms from environments.

In summary, the Tibetan hot springs host unique HPB populations, the *hydA* genes affiliated with *Bacteroidetes* and *Spirobacteria* dominated the studied samples, but that affiliated with *Proteobacteria* was lacking. The HPB community structure was correlated with pH, temperature, elevation, and redox potential in the studied Tibetan hot springs. In addition, HPB strain QZM-1 was successfully isolated from the hot spring sediments in Quzhuomu area, and its *hydA* gene and function of hydrogen production were confirmed. These results are important for us to understand the distribution and function of HPB in hot springs.

## Data Availability Statement

The datasets presented in this study can be found in online repositories. The names of the repository/repositories and accession number(s) can be found in the article/Supplementary Material.

## Author Contributions

GW and HJ conceived the study. LM, JY, DP, and GW performed on-site measurements and collected the samples. LM, JY, DP, W-JL, and GW analyzed geochemistry and microbiology of the samples. LM, LH and JY analyzed the sequencing data. LM, GW, and HJ drafted the manuscript. All authors reviewed results and commented on the manuscript.

## Conflict of Interest

The authors declare that the research was conducted in the absence of any commercial or financial relationships that could be construed as a potential conflict of interest.

## Footnotes

1 http://www.usadellab.org/cms/?page=trimmomatic

2 https://www.arb-silva.de/

3 https://ftp.ncbi.nlm.nih.gov/blast/db/FASTA/

4 http://cran.r-project.org/
