# Supplementary material for: Distribution of Hydrogen-Producing Bacteria in Tibetan Hot Springs, China
Source: Front Microbiol. 2021 Jul 21;12:569020. doi: 10.3389/fmicb.2021.569020 (PMC8334365; doi:10.3389/fmicb.2021.569020)
Supplement: Supplementary file 1 [file Presentation_1.pdf]

Supplementary figures and tables:

Figure S1 Distribution of *hydA* in sediment sampled from 66 geothermal springs locations in Tibetan hot springs as a function of spring water pH and temperature. Orange triangles denote environments where amplicons were detected and blue squares denote environments where amplicons were not detected

Figure S2 Pearson's rank correlations between the measured environmental variables and HPB population relative abundance

Figure S3 Autocorrelation test results of environmental factors

Figure S4 A neighbor-joining phylogenetic tree of 16S rRNA sequence (A) and HydA amino acid sequence (B) was constructed with the Poisson model in the MEGA 7.0 software.

Figure S5 Hydrogen production verification of Strain QZM-1

Table S1. Ecological estimates of the hydA gene of the investigated hot spring sediments in this study

Figure S1 Distribution of hydA in sediment sampled from 66 geothermal springs locations in Tibetan hot springs as a function of spring water pH and temperature. Orange triangles denote environments where amplicons were detected and blue squares denote environments where amplicons were not detected

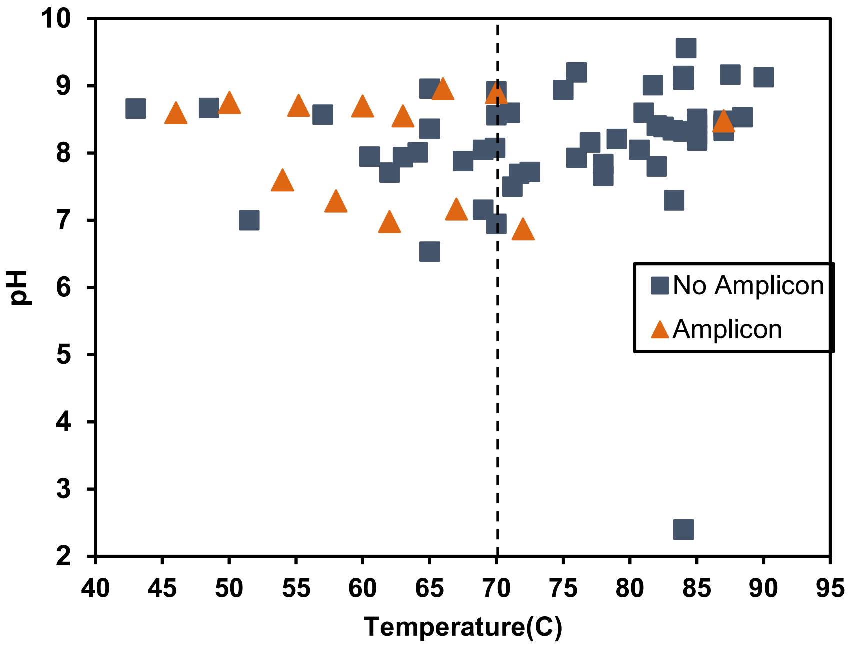

Figure S2 Pearson's rank correlations between the measured environmental variables and HPB population relative abundance

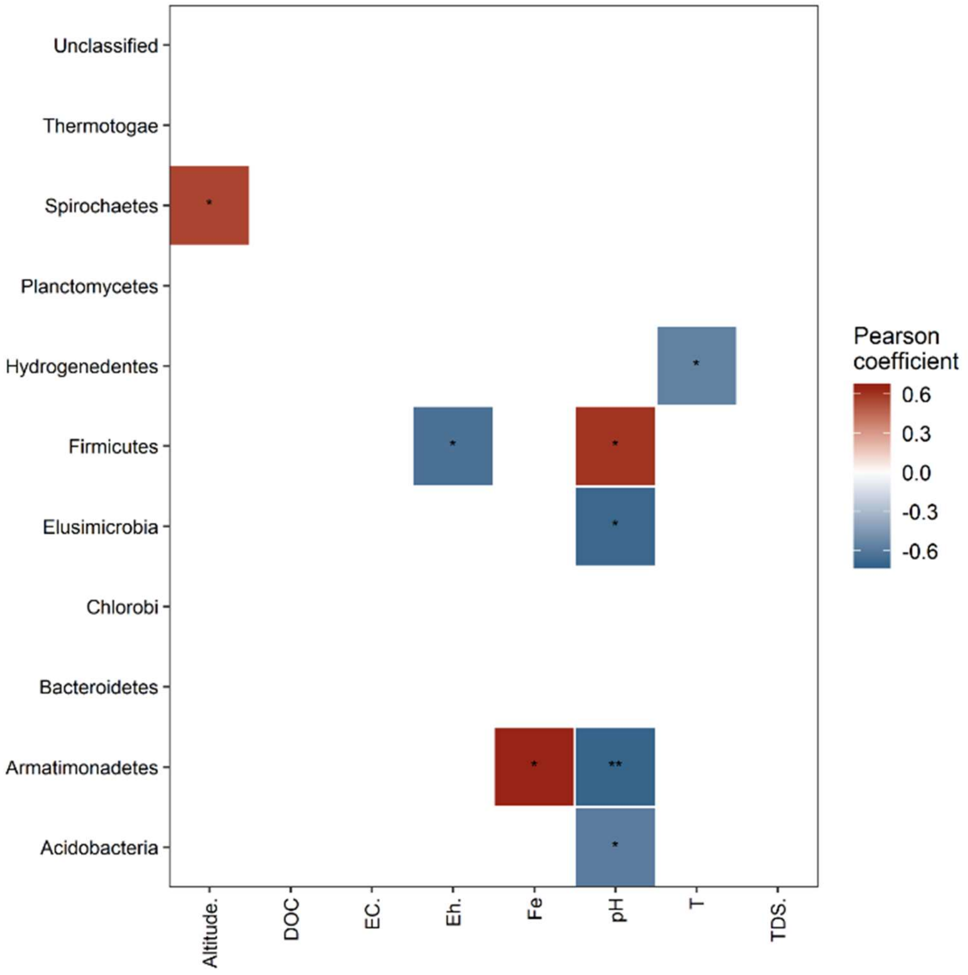

("*": p<0.05;"**": p<0.01;"***": p<0.001).

Figure S3 Autocorrelation test results of environmental factors

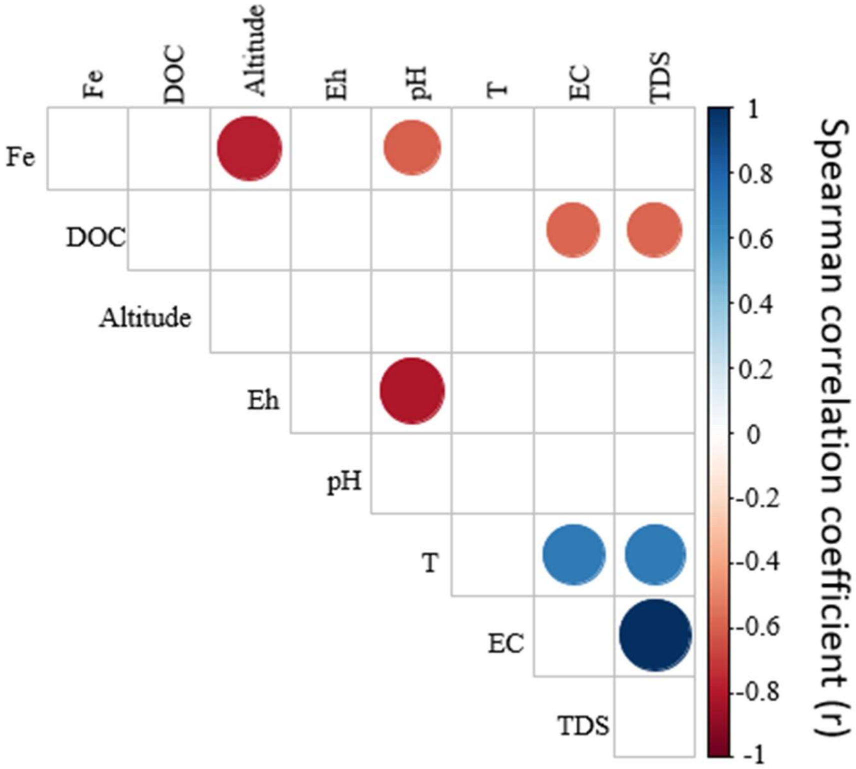

Figure S4 Maximum likelihood phylogeny of the 16S rRNA sequence (A) and *HydA* amino acid sequence (B) of the isolate obtained from the investigated hot springs was constructed with the Poisson model in the MEGA 7.0 software.

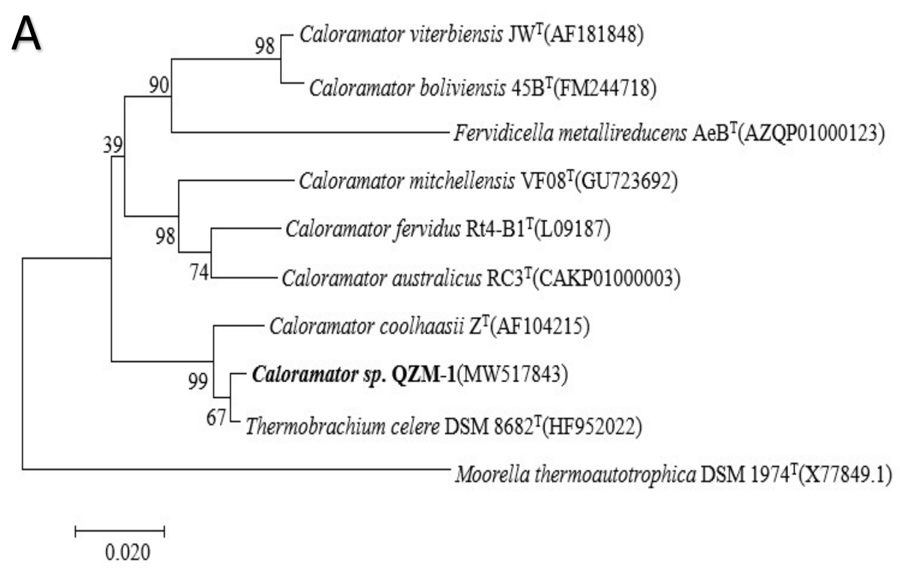

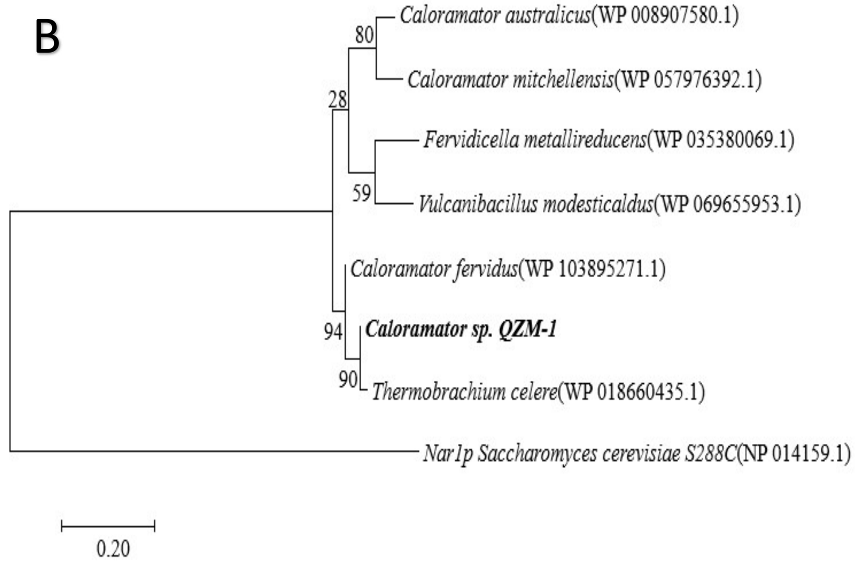

Figure S5 Hydrogen production verification of Strain QZM-1

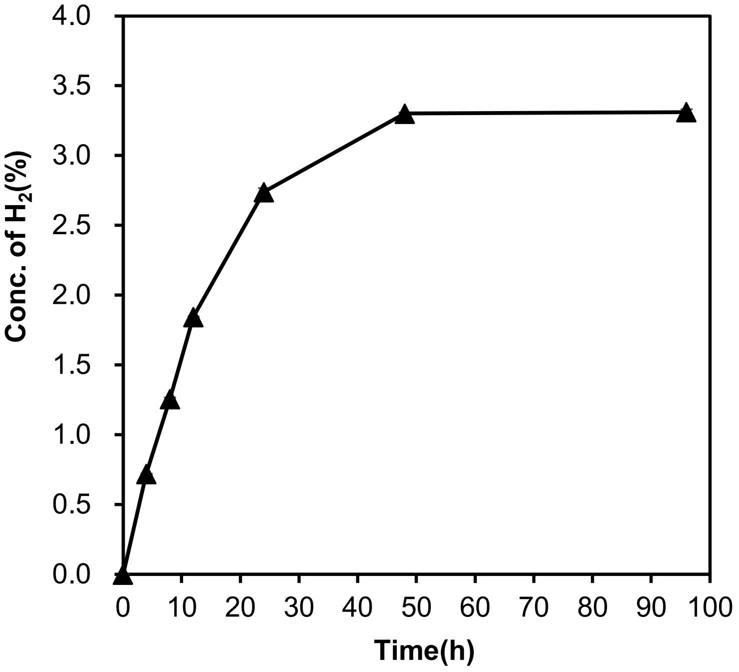

Table S1. Ecological estimates of the *hydA* gene of the investigated hot spring sediments in this study

Sample	Number Reads	Observed OTUs	Shannon Wiener	Simpson
QSYB09-3	61260	480	4.195	0.945
QSYB09-4	69147	353	2.447	0.776
MLJ-5	80021	422	3.281	0.898
DG01-3	87372	286	3.525	0.932
DG01-4	91124	199	3.139	0.923
DG01-5	82724	215	2.939	0.888
DG01-6	74253	246	3.660	0.949
DG02-2	67311	96	2.371	0.830
QZM04	59323	151	2.893	0.891
QZM04-1	76023	205	2.686	0.876
QZM04-2	65556	217	2.958	0.875
QZM04-3	69866	189	3.630	0.951
QZM04-4	74025	267	3.931	0.964
